# End-to-end vascular anastomosis using a novel magnetic compression device in rabbits: a preliminary study

**DOI:** 10.1038/s41598-020-62936-6

**Published:** 2020-04-06

**Authors:** Qiang Lu, Kang Liu, Wei Zhang, Tao Li, Ai-Hua Shi, Hong-Fan Ding, Xiao-Peng Yan, Xu-Feng Zhang, Rong-Qian Wu, Yi Lv, Shan-Pei Wang

**Affiliations:** 1 National Local Joint Engineering Research Center for Precision Surgery & Regenerative Medicine, Xi’an, Shaanxi Province China; 2 Shaanxi Provincial Center for Regenerative Medicine and Surgical Engineering, Xi’an, Shaanxi Province China; 3 Institute of Advanced Surgical Technology and Engineering, Xi’an, Shaanxi Province China; 4 Department of Hepatobiliary Surgery, First Affiliated Hospital of Xi’an Jiaotong University, Xi’an, Shaanxi Province China

**Keywords:** Preclinical research, Translational research

## Abstract

Magnetic compression anastomosis (MCA) has been appreciated as an innovative alternative to manual suturing in vascular reconstruction. However, magnetic devices have limitations in their applications. The present study aimed to introduce a newly developed magnetic device for end-to-end vascular anastomosis. Twenty male New Zealand rabbits were randomly assigned to receive end-to-end postcaval vein anastomosis using either a newly designed MCA device (Group MCA) or continuous-interrupted suturing (Group CIS). The anastomotic patency was evaluated by Doppler or venography immediately, 1 week, and 12 weeks after surgery. Anastomotic quality was evaluated gross and microscopic histological study 12 weeks after surgery. The procedure was successfully performed and all animals survived until sacrifice. The duration of surgery and anastomosis time in Group MCA were significantly shorter compared to Group CIS (all p < 0.001), and the incidence of anastomotic patency and postoperative morbidity were comparable between the two groups (all p > 0.05). Hematoxylin-eosin staining showed that anastomotic intima from Group MCA was much smoother with more regularly arranged endothelial cells than from compared to the Group CIS. A novel MCA device was successfully applied in rabbit vascular anastomosis. We demonstrated the reliability and effectiveness of this newly developed MCA in this study.

## Introduction

Vascular anastomosis, commonly performed in complicated surgical procedures (e.g. transplantation, reconstruction, etc.), is a sophisticated technique that requires advanced skills^1–3^. The quality and reliability of the technique, to a great extent, determine the success of the procedure. Currently, the standard method for vascular anastomosis is a handheld suture technique originally described by Carrel in 1902^4^. However, to be competent for this task requires specialized training, which is time-consuming and involves a steep learning curve, and the technique itself is likely subject to human error. Even for highly skilled surgeons, anastomosis-related complications (e.g. anastomotic leak, stenosis, thrombosis, etc.), mainly due to vessel damage or blood flow turbulence generated by the suture, occur approximately once in every 10 cases^5–7^.

Sutureless anastomosis has been considered as an ideal alternative for manual suturing in vascular reconstruction. So far, techniques and devices have been developed for sutureless repair of vessels^8–12^. Magnetic compression anastomosis (MCA), firstly described by Obora *et al*. in 1978^13^, is a promising sutureless anastomotic technique for vascular anastomosis. This technique has been successfully applied for end-to-end and side-to-side anastomoses in animal experiments^14–19^. Unfortunately, there were shortcomings with the magnetic devices used in previous studies, including difficulty in installation, disability in microvascular anastomosis, and potential hazard to performers. In the present study, we report a newly developed MCA device for end-to-end vascular anastomosis.

## Materials and Methods

### Magnetic devices

**Figure 1:**
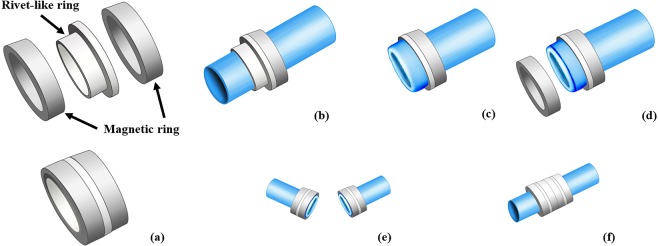
Working mechanism of the magnetic compression anastomotic devices: (**a**) one set of the device consists of 2 magnetic rings and 1 rivet-like ring; (**b**) allow one vessel end to pass through the magnetic ring/rivet-like ring complex; (**c**) evert the end of the vessel to cover the cylindrical structure of the rivet-like ring; (**d**) apply the paired magnetic ring onto the cylindrical structure of the rivet-like ring and allow magnetic attraction to keep the set as a whole; (**e**) install the other end of the vessel following the same steps; and (**f**) simply connect the two ends.

**Figure 2:**
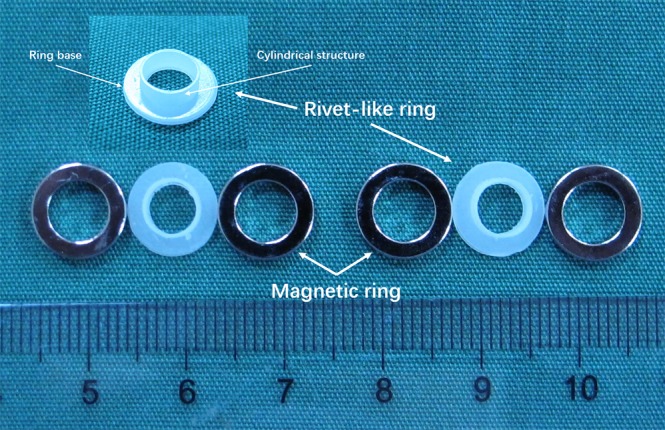
Magnetic compression device for end-to-end vascular anastomosis.

The magnetic device employed in the present study consisted of two pairs of magnetic rings and two rivet-like rings (See Fig. 1). The magnetic rings were made of sintered neodymium-ferrum-boron materials (Ti-NdFeB). The parameters of the magnetic rings were: inner diameter 6 mm, outer diameter 9 mm, thickness 1 mm, and weight 0.35 g (See Fig. 2). The rivet-like rings were made of Polyetheretherketone (PEEK) generated by 3D printing, and consisted of a ring base and a cylindrical structure with a smooth inner surface to prevent vessel damage (Fig. 2). The ring base had a diameter of 9 mm, while the parameters of the cylindrical structure were: outer diameter 5.8 mm, inner diameter 4.8 mm, and height 1 mm. This structure allowed the cylindrical part to slide into the magnetic ring (Fig. 1a). The working mechanism of the magnetic device is illustrated in Fig. 1b-f.

### Animals and grouping

The study was conducted in strict accordance with the recommendations of the Xi’an Jiaotong University Medical Center Guide for the Care and Use of Laboratory Animals. The protocol has been approved by the Animal Experiment Ethics Committee of Xi’an Jiaotong University (Permit Number 2010-105).

Twenty male New Zealand white rabbits weighing from 3.5 to 4.0 kg were used in this study. The rabbits were randomly assigned to two groups to undergo end-to-end postcaval vein anastomosis by different techniques: in Group MCA (n = 10) using the magnetic device, and in Group CIS (n = 10) using continuous-interrupted suture^20^. All rabbits received antibiotic prophylaxis of cefazolin sodium (10.0 mg/kg) by subcutaneous injection before the procedure. For anesthesia, the rabbits were administered 0.1 mg/kg medetomidine and 5 mg/kg of carprofen subcutaneously, followed by the induction of intravenous sufentanil (2.3 ug/kg) and midazolam (0.2 mg/kg). After endotracheal intubation, anesthesia was maintained using sufentanil-midazolam. The rabbits were placed in a supine position to expose the abdomen region well. The surgical area was then shaved, sterilized with povidone iodine, and draped with sterile towels.

### Surgical procedure and technique

**Figure 3:**
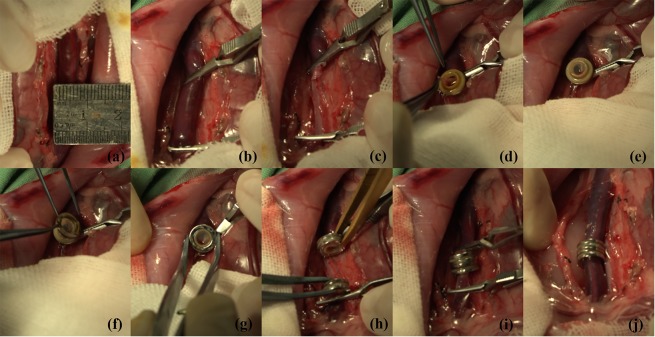
Process of anastomosis: (a) exposure of the postcaval vein; (**b**) clamping of the postcaval vein; (**c**) disconnection of the postcaval vein; (**d**) one end of the postcaval vein is passed through the rivet-like ring/magnetic ring complex; (**e**) eversion of the vessel end; (**f**,**g**) assembly of the paired magnetic ring; (**h**) completion of the magnetic devices installment on the other end; (**i**) end-to-end vascular anastomosis done by mutual attraction of two magnetic sets; and (**j**) recanalization of the postcaval vein.

The procedure started with an approximate 7 cm longitudinal incision made along the linea mediana ventralis. The postcaval vein was exposed after the posterior peritoneum was cut open, and a 4–5 cm segment of the postcaval vein was isolated (See Fig. 3a). Vein hemostatic clips were applied to the proximal and distal ends of where the anastomosis was going to be (See Fig. 3b). Then, the postcaval vein was carefully disconnected, and the vessel stump lumen was rinsed thoroughly with a heparin-saline solution (500 U heparin/mL) to remove residual blood in order to prevent thrombosis (See Fig. 3c). End-to-end postcaval vein anastomosis was then performed in each rabbit. In Group MCA, the surgeon followed the steps summarized as follows. First, one of the vessel ends was allowed to pass through a magnetic ring, followed by the rivet-like ring in turn, leaving approximately 2 mm of the vessel end beyond the edge of the ring (See Fig. 3d). The vessel end was then everted using titanium alloy vascular tweezers (See Fig. 3e). The paired magnetic ring was installed onto the rivet-like ring by sliding the rivet-like ring into the magnetic ring (See Fig. 3f,g). The same installation procedure was repeated on the other vessel end (See Fig. 3h). Finally, the magnetic-device-installed vessel ends were connected to complete the end-to-end vascular anastomosis (See Fig. 3i). The hemostatic clips were then released to restore blood flow (See Fig. 3j). In Group CIS group, anastomosis was performed using the continuous-interrupted suture technique with 7–0 Prolene. The abdominal incision was closed with two-layer interrupted 3–0 nylon sutures. The same surgeon performed both the MCA and CIS techniques.

### Postoperative management

Body temperature, heart rate, and arterial oxygen saturation were monitored using a veterinary monitor after the surgery in all rabbits until all animals awoke. Daily antibiotic (cefazolin sodium, 10.0 mg/kg, twice a day) and analgesic therapy (Phenylbutazone, 10 mg/kg, once a day) were continued for 3 days after the surgery. No anticoagulant therapy was given to the rabbits in either group after the surgery.

### Imaging study

Abdominal vascular ultrasound (ACUSON X 150 Diagnostic Ultrasound System, Siemens Medical Solutions Inc., USA) was performed to evaluate the patency of postcaval vein anastomotic stoma immediately, 1 week, and 12 weeks after surgery. Velocity ratio > 2.5 (post-stenotic to pre-stenotic vein velocity ratio) during ultrasound examination was considered vascular obstruction^21^. Transfemoral venography (PLX7000B High Frequency Mobile C arm System, Nanjing Perlove Medical Equipment Co., Ltd., China) was also employed as an alternative if vascular ultrasound failed.

### Tissue harvest and histological study

At the end of the week 12 after the surgery, the rabbits were sacrificed by cardiac injection of potassium chloride after evaluating anastomotic patency, and the venous tissues, including the anastomotic site in both groups, were sampled for histopathological analysis. All samples were fixed in 10% neutral buffered formalin. Longitudinal paraffin sections were cut at a 5 μm thickness and stained with hematoxylin and eosin (HE). Masson’s trichrome stain was used to differentiate the connective tissue components.

### Statistical analysis

Data are presented as mean values ± standard deviation. Differences between means were assessed using the paired t-test or Wilcoxon signed rank test where applicable. A P-value less than 0.05 was considered statistically significant.

## Results

### Surgical results

The end-to-end anastomosis of the postcaval vein was successful in both study groups. All rabbits survived after the surgery and recovered without anastomotic bleeding. Comparisons of major parameters using two different anastomotic techniques are shown in Table 1. The body weight and postcaval vein diameter were similar between the two groups (p > 0.05). As expected, time of the whole procedure and of anastomosis in Group MCA were significantly shorter compared to Group CIS (52 ± 5 min vs 81 ± 3 min and 10 ± 1 min vs 29 ± 4 min, respectively; both p < 0.001). No anastomotic obstruction or stenosis was observed in Group MCA, while anastomotic stenosis was detected in 1 rabbit and anastomotic obstruction was found in another rabbit from Group CIS. However, no difference was found in anastomotic obstruction or stenosis between the two groups (All p > 0.05).

**Table 1:** Comparisons of the peri-operative data between Group MCA and Group CIS.

	MCA (n = 10)	CIS (n = 10)	p
Body weight (kg)	3.8 ± 0.2	3.8 ± 0.2	0.42
Postcaval vein diameter (mm)	5.5 ± 0.7	6.0 ± 0.8	0.57
Operation time (min)	52 ± 5	81 ± 3	<0.001
Anastomosis time (min)	10 ± 1	29 ± 4	<0.001
Patency at 12 weeks (n, %)	10 (100%)	9 (90%)	1.00
Anastomotic stenosis (n, %)	0 (0)	1 (10%)	0.47

### Ultrasonographic results

**Figure 4:**
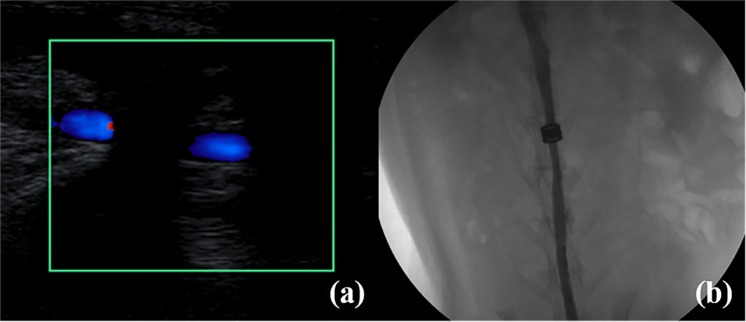
Representative ultrasound and angiographic images after surgery. (**a**) Ultrasound study of the vascular anastomoses 12 weeks after surgery; (**b**) postcaval vein angiography 12 weeks after surgery.

No obstruction or leak in postcaval venous inflow was found in experimental rabbits immediately after the surgery. No anastomotic obstruction or stenosis was detected in in Group MCA at the twelfth week after surgery (See Fig. 4a,b). At the same time point, anastomotic stenosis was observed in 1 rabbit, and anastomotic obstruction was found in another rabbit in Group CIS.

### Histological results

**Figure 5:**
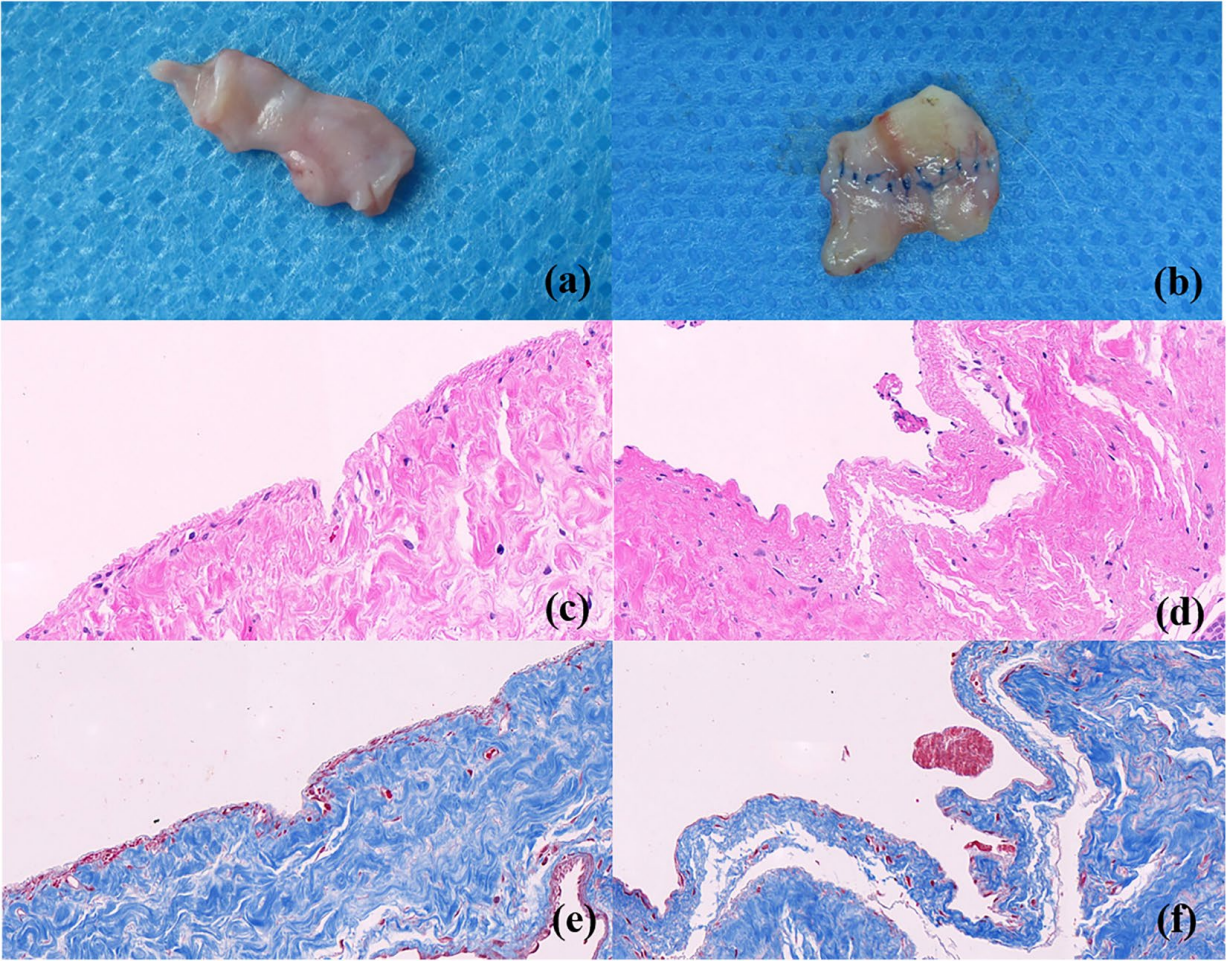
Gross appearance and histology of vascular anastomosis 12 weeks after surgery. (**a**,**b**) Gross views of the end-to-end portacaval anastomoses from Group MCA (**a**) and Group CIS (**b**); histological analysis of anastomosis: HE stained × 40, Group MCA (**c**) and Group CIS (**d**); Masson stained × 40, Group MCA (**e**) and Group CIS (**f**).

Significant differences were observed between the two groups in terms of gross appearance of the anastomotic specimens (see Fig. 5). In Group MCA, the magnetic device was macroscopically covered by a thin layer of fibrous tissue capsule, and the endovascular surface of the anastomotic stoma was covered with layers of intima and appeared to be completely endothelialized (see Fig. 5a,c,e). In addition, no foreign body remained in the lumen of the vessel. However, in Group CIS, the sutures were visible from the internal aspect of the anastomosis, and the inner surface of the anastomosis was rough and uneven (see Fig. 5b,d,f).

Representative images of the HE-stained anastomotic stomas retrieved from the two groups were obtained at 12 weeks after the surgery. In Group MCA, there was no rough surface or obvious fibrin clot on the surface of the blood vessels. A neat intima-to-intima match was observed and vascular anastomotic endothelial cells and collagen fibers arranged regularly. However, in Group CIS, the neointima arrangement was integrated but irregular, and the vascular collagen fiber layers were distorted.

## Discussion

MCA, as a surgical technique, was firstly introduced by Obora *et al*. in 1978. In the past few decades, this technique has been proved successful in both side-to-side and end-to-end vascular anastomoses in previous animal experiments. It has been considered as an optimal alternative approach to manual suturing in vascular reconstruction. In previous studies, Yan and Xue^15,18,19^ used a specially designed magnetic device to perform minimally invasive portal shunt surgery in a portal hypertension animal model. Liu *et al*.^22^ designed a magnetic pinning-ring device for end-to-end vascular anastomosis and successfully applied it during liver transplantation in an animal study. In addition, Wang *et al*.^16^ employed a new type of MCA device to complete fast magnetic reconstruction of the portal vein with allogeneic blood vessels in canines. However, There are no magnetic devices for microvascular anastomosis, especially for small vein anastomosis.

The newly developed magnetic device used in the present study was successfully demonstrated as an effective and reliable approach for end-to-end vascular anastomosis in rabbits. Compared to the previous device, the MCA we used in this study has shown impressive advantages, which enable it to better aid the performer in vascular anastomosis. Obviously, it is easy-operating, creates neat intima-to-intima contact anastomosis, and minimize trauma to the vascular endothelium. The MCA used in this study allows the blood vessel to be everted by clicking a magnetic ring onto the cylindrical structure of a PEEK-made rivet-like ring, instead of being penetrated by a circle of minor pins. This allows the process to be simpler and safer, as the risk of occupational exposure of the surgeon is eliminated. Since the magnetic ring/rivet-like ring complex is placed in the middle, the vessel end is less tensive, which reduces the potential of vessel-end tearing. In addition, the vessel end is firmly fixed to the device once the parent and daughter magnetic rings attract each other, which prevents the device from falling off the vessel end. The magnetic device on both vascular stumps can be rotated to correct torsion, even after the two installed ends are connected to each other. Furthermore, the device is considered biocompatible. The surface of the magnetic ring is coated with titanium, a metal which has been extensively used as implant materials with great biocompatibility^23^, and the rivet-like ring is made of PEEK, which is also highly biocompatible^24^. The force required to pull the magnetic devices apart is 7 N in this study, which is sufficient to prevent the anastomosis stoma from getting apart for the venous pressure is very low.

There were several limitations of this study. Although this animal study successfully demonstrated the reliability and efficiency of our newly developed magnetic device in vascular anastomosis in rabbits, further experiments in larger mammals are required to evaluate the feasibility and safety before any human clinical trials can be conducted. Second, the postoperative observation time of this study is short, which did not allow the possibility of assessing long-term outcomes. Moreover, the effect of the magnetic field of the device on hemodynamics was not evaluated in the study. Previous studies have found that the magnetic field affects the orientation of normal red blood cells^25,26^. Therefore, it is highly possible that when it flows through the anastomotic site, blood may be affected by the magnetic field, which may lead to the occurrence of anastomotic complications. However, the incidence of postoperative anastomotic-related complications was comparable between Group MCA and Group CIS in this study during follow-up. What’ s more, MRI is still limited in animals or patients who had undergone magnetic compression anastomosis^27^. Following studies are still needed to further evaluate the safety of animals or patients with magnetic devices during MRI inspection. In addition, this magnetic device was not tested in superficial vein, and the burst pressure of the anastomotic stomas was also not measured in this study. However, previous research on magnetic compression anastomosis in rapid vascular reconstruction has indicated that magnetic ring anastomotic stomas were able to withstand over 280 mmHg^22^. At the same time, it is unknown whether this magnetic device will function on arteries. However, another magnetic devic had been applied in superficial artery anastomosis^28^.

Based on previous research experience with MCA, we designed a new type of magnetic device for end-to-end vascular anastomosis, and successfully demonstrated its reliability and effectiveness in end-to-end postcaval vein anastomosis in rabbits. The promising results of the present study encourage future evaluation of magnetic vascular anastomosis in larger mammal studies.
