# Author Correction: End-to-end vascular anastomosis using a novel magnetic compression device in rabbits: a preliminary study

**DOI:** 10.1038/s41598-020-73419-z

**Published:** 2020-10-02

**Authors:** Qiang Lu, Kang Liu, Wei Zhang, Tao Li, Ai-Hua Shi, Hong-Fan Ding, Xiao-Peng Yan, Xu-Feng Zhang, Rong-Qian Wu, Yi Lv, Shan-Pei Wang

**Affiliations:** 1 Department of Hepatobiliary Surgery, First Affiliated Hospital of Xi’an Jiaotong University, Xi’an, Shaanxi China; 2 National Local Joint Engineering Research Center for Precision Surgery & Regenerative Medicine, First Affiliated Hospital of Xi’an Jiaotong University, Xi’an, Shaanxi China; 3 Shaanxi Provincial Center for Regenerative Medicine and Surgical Engineering, First Affiliated Hospital of Xi’an Jiaotong University, Xi’an, Shaanxi China; 4 Institute of Advanced Surgical Technology and Engineering, First Affiliated Hospital of Xi’an Jiaotong University, Xi’an, Shaanxi China

Correction to: *Scientific Reports* https://doi.org/10.1038/s41598-020-62936-6, published online 06 April 2020

The original version of this Article contained errors in Affiliations 1, 2, 3 and 4, which were incorrectly listed as ‘National Local Joint Engineering Research Center for Precision Surgery & Regenerative Medicine, Xi’an, Shaanxi Province, China’, ‘Shaanxi Provincial Center for Regenerative Medicine and Surgical Engineering, Xi’an, Shaanxi Province, China’, ‘Institute of Advanced Surgical Technology and Engineering, Xi’an, Shaanxi Province, China’ and ‘Department of Hepatobiliary Surgery, First Affiliated Hospital of Xi’an Jiaotong University, Xi’an, Shaanxi Province, China’, respectively.

The correct affiliations are listed below.

Affiliation 1:

Department of Hepatobiliary Surgery, First Affiliated Hospital of Xi'an Jiaotong University, Xi'an, Shaanxi Province, China

Affiliation 2:

National Local Joint Engineering Research Center for Precision Surgery & Regenerative Medicine, First Affiliated Hospital of Xi'an Jiaotong University, Xi'an, Shaanxi Province, China

Affiliation 3:

Shaanxi Provincial Center for Regenerative Medicine and Surgical Engineering, First Affiliated Hospital of Xi'an Jiaotong University, Xi'an, Shaanxi Province, China

Affiliation 4:

Institute of Advanced Surgical Technology and Engineering, First Affiliated Hospital of Xi'an Jiaotong University, Xi'an, Shaanxi Province, China

This error has now been corrected in the PDF and HTML versions of the Article.

